# Genomic Insights and Conservation Priorities for Kongshan Cattle: A Whole-Genome Resequencing Approach

**DOI:** 10.3390/ani14213056

**Published:** 2024-10-23

**Authors:** Wenqiang Sun, Hanjun Ren, Mengze Li, Liping Mei, Bingfei Zhang, Xianbo Jia, Shiyi Chen, Jie Wang, Songjia Lai

**Affiliations:** 1 Farm Animal Genetic Resources Exploration and Innovation Key Laboratory of Sichuan Province, College of Animal Science and Technology, Sichuan Agricultural University, Yaan 625014, China; wqsun2021@163.com (W.S.); 17392547544@163.com (H.R.); 15706065790@163.com (M.L.); 15700312879@163.com (L.M.); zhangbingfei07@163.com (B.Z.); jaxb369@sicau.edu.cn (X.J.); chensysau@163.com (S.C.); wjie68@163.com (J.W.); 2 State Key Laboratory of Swine and Poultry Breeding Industry, College of Animal Science and Technology, Sichuan Agricultural University, Yaan 625014, China; 3 Key Laboratory of Livestock and Poultry Multi-Omics, Ministry of Agriculture and Rural Affairs, College of Animal Science and Technology, Sichuan Agricultural University, Yaan 625014, China

**Keywords:** Kongshan cattle, whole-genome resequencing, SNPs, small indel, functional annotation

## Abstract

Kongshan Cattle, indigenous to Sichuan Province and recognized as China’s 56th local cattle breed in 2024, exhibit unique adaptations including superior resistance to harsh conditions. Despite a declining population due to the influx of foreign breeds, there is a significant focus on preserving their genetic diversity through advanced genomic techniques. This study utilized whole-genome resequencing, a cost-effective and information-rich method, to perform a comprehensive genetic assessment of the Kongshan Cattle. High-quality resequencing data yielded an average of 17.5 billion clean bases per sample, with high proportions of Q20 and Q30 bases and a balanced GC content. SNP analysis revealed an average of 14 million SNPs per sample, with a notable transition-to-transversion ratio and a significant portion of heterozygosity. Further analysis of genomic and coding regions identified substantial insertions and deletions, particularly in coding sequences affecting gene functionality. A detailed examination of these genetic variations highlighted genes, including NEIL2 and PNKP, which are integral to stress resistance pathways, indicating potential adaptive advantages. This study not only underscores the genetic diversity of Kongshan Cattle but also contributes to broader efforts in germplasm conservation.

## Simple Summary

Kongshan Cattle, a breed native to Sichuan Province, China, recognized for their resilience to adverse conditions, are the focus of a critical conservation effort due to their declining numbers. This study used whole-genome resequencing to analyze their genetic structure comprehensively. The resequencing data revealed an average of 17.5 billion clean bases per sample, demonstrating high data quality with significant SNP discoveries—approximately 14 million SNPs per sample. We also identified key gene variants that could contribute to the breed’s unique traits, such as their noted stress resistance. These findings are crucial for future conservation strategies and highlight the importance of preserving the genetic diversity of local cattle breeds like Kongshan Cattle.

## 1. Introduction

Kongshan Cattle, native to Tongjiang County in Sichuan Province, were officially recognized in 2024 as China’s 56th local cattle breed by the National Livestock Genetic Resources Committee. They exhibit superior resistance to moisture and adverse conditions, outperforming cattle from plains and hilly areas, and have fewer diseases [1]. Although Kongshan Cattle are slender and produce less meat, their meat quality aligns well with the preferences of Chinese consumers, making them an integral part of China’s cattle biodiversity [1]. The population of Kongshan Cattle sharply declined in the late 20th century following the introduction of large quantities of foreign cattle breeds for domestic improvement. Given the urgency to protect such valuable local breeds, in-depth research and analysis of their genetic diversity and structure are necessary.

The advent of advanced sequencing technologies, particularly second-generation techniques, like high-throughput sequencing, resequencing, de novo sequencing, and exome sequencing, now represent the most widely used and effective methods in genomics [2]. Resequencing, being both cost-effective and rich in genetic information, plays a critical role in analyzing the genetic structure of species [3]. This technique has been extensively utilized in cattle genetic studies, as demonstrated by Naveed Iqbal et al. [4], Elisa Peripolli et al. [5], and Shunjin Zhang et al. [6], who have identified genomic variants and signatures of selection in various cattle breeds through whole-genome resequencing.

Given the significant research potential of Kongshan Yellow Cattle, and the lack of comprehensive genomic data available for this breed, analyzing their whole genome to assess genetic diversity and structure could stabilize their genetic foundation. Thus, this research not only provides a scientific basis for genome studies and functional gene screening for germplasm conservation but also aids in establishing complete pedigree data and stabilizing the genetic characteristics of Kongshan Yellow Cattle.

## 2. Materials and Methods

### 2.1. Sample Collection, DNA Extraction and Sequencing

Blood samples were collected from 40 Kongshan cattle by licensed veterinarians in Tongjiang County, Bazhong City, Sichuan Province, China. The samples were drawn from the jugular vein under sterile conditions using 10 mL EDTA tubes to ensure the integrity and quality of the samples for genomic analysis. Total genomic DNA was extracted using the standard procedure provided by an Animal Genomic DNA Kit (Tiangen, Beijing, China). DNA quality and quantity were assessed using NanoVue Plus (GE, USA). DNA libraries (350 bp) suitable for Illumina/BGI sequencing were prepared following the manufacturer’s specifications. Sequencing was conducted on an Illumina HiSeq XTen/NovaSeq/BGI platform by Biomarker Technologies (Beijing, China), generating 150 bp reads. Raw reads were filtered to exclude pair-end reads with >10% “N” bases and reads where over 50% of bases had a quality score below 20 (Phred-like score). After adapter removal, high-quality sequences were retained for analysis.

### 2.2. SNP and InDel Calling

All clean reads were aligned to the reference genome using the MEM algorithm of Burrows–Wheeler Aligner (bwa-mem2 v2.2). The aligned reads were sorted and duplicates removed using samtools (v1.7) [7]. Subsequently, samtools (v1.7) was utilized to sort the aligned reads and remove duplicates, ensuring data integrity for downstream analysis [8] and filtered by parameters including QD < 2.0, MQ < 40.0, FS > 60.0, QUAL < 30.0, MQrankSum < −12.5, and ReadPosRankSum < −8.0. Following further filtration, SNP annotation was conducted using snpEff (v3.6c) [9], categorizing SNPs into regions such as intergenic, upstream/downstream, and coding (synonymous or nonsynonymous). InDels in coding exons were classified by their potential to cause frameshift mutations.

### 2.3. Functional Analysis of Variant Genes

Gene Ontology (GO) and Kyoto Encyclopedia of Genes and Genomes (KEGG) pathway enrichment analyses on variant genes were performed using the Database for Annotation, Visualization and Integrated Discovery (DAVID 2021) [10,11].

### 2.4. Statistical Analyses

Data were analyzed via one-way ANOVA in SAS 9.0, with Duncan’s method for post hoc comparisons. Results were presented as mean ± SD, with significance indicated by * *p* < 0.05.

## 3. Results

### 3.1. Comprehensive Quality Assessment of Resequencing Data for Kongshan Cattle

After performing quality checks and filtering on the raw data, each sample of Kongshan Cattle yielded an average of 17.5 billion high-quality bases (clean bases). Among these, the proportions of Q20 and Q30 bases were notably high at 97.88% and 94.43%, respectively, while the GC content was balanced at 42.71%. These results indicate a high quality of sequencing data, with the base quality and GC content showing no significant deviations (Table 1).

### 3.2. Genomic SNP Variant Analysis: Transition, Transversion, and Zygosity Rates

Quality control filtering of mutation sites revealed an average of 14,058,387.00 SNPs per sample. Of these, 9,957,241.00 were transition-type SNPs and 4,101,146.00 were transversion-type SNPs, resulting in a transition-to-transversion ratio of 2.42. The count of heterozygous SNPs was 3,266,503.78, while the number of homozygous SNPs was 10,791,883.23, constituting 23.19% of the total SNPs as heterozygous (Table 2).

### 3.3. Analysis of Fragment Insertions and Deletions (InDels) in Genomic and Coding Regions

**Figure 1:**
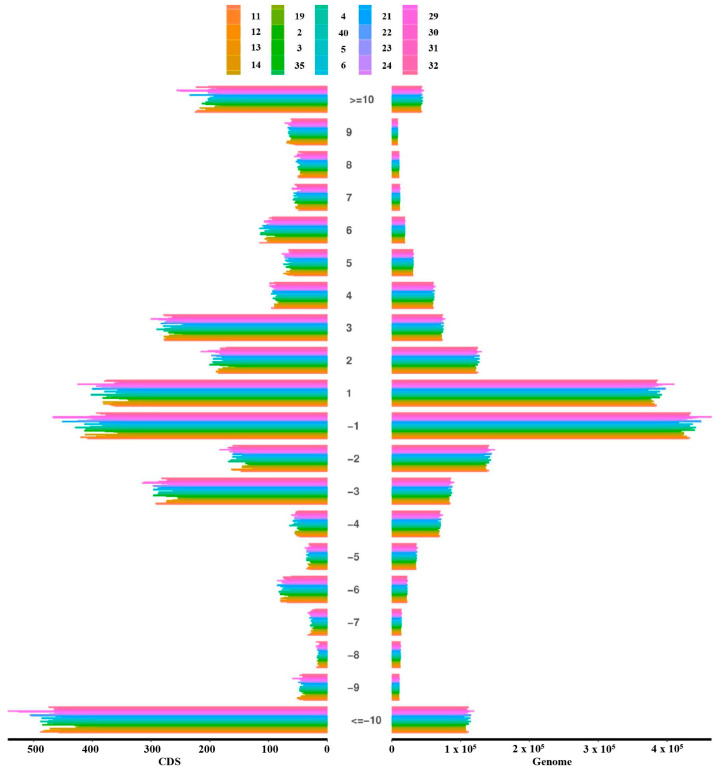
Genome-wide and coding region InDel length distribution.

Quality control filtering identified a total of 1,818,975 sites with fragment insertions and 2,987,128 sites with fragment deletions. Among these, there were 4026 coding region sites with insertions and 6225 with deletions (Table 3). Additionally, the highest number of deletions in coding regions involved fragments more significant than 10 bp, followed by insertions and deletions of 1 bp fragments. In the genomic regions, sites with 1 bp insertions and deletions were most frequent, followed by sites with deletions of fragments larger than 10 bp (Figure 1). Additionally, we performed an analysis of all differential variants between samples, which is summarized in Table S1, and an analysis of all differential small InDels between samples, as detailed in Table S2. These analyses highlight the diverse genomic landscape of the population studied

### 3.4. Mining Genetic Variations at the DNA Level

**Figure 2:**
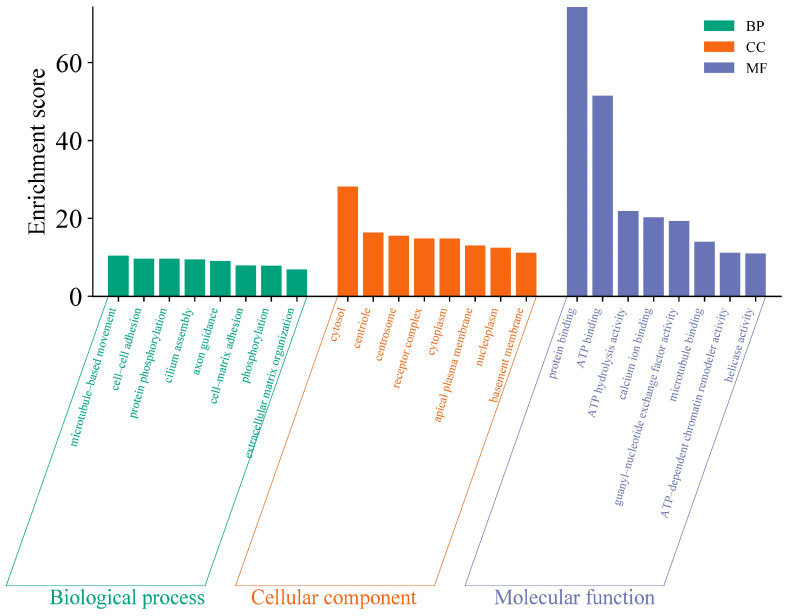
Gene ontology (GO) analysis of part of variant genes.

**Figure 3:**
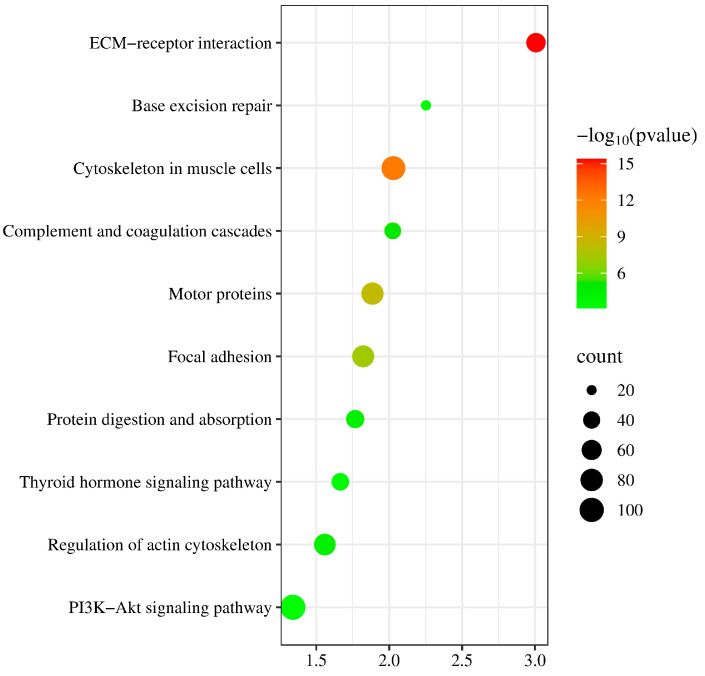
Kyoto Encyclopedia of Genes and Genomes (KEGG) pathway analysis of part of variant genes.

Due to mutations in the coding sequence (CDS) regions affecting gene functionality, this study compiled statistics on the differential genes caused by mutations, as shown in Table 4. The average number of genes with non-synonymous SNPs was 14,302.38, and those with insertions or deletions averaged 3278 (Table S3). To further explore the functions of these variant genes, we first overlapped all variant genes across 40 individuals, revealing that 4873 known genes exhibited mutations in all individuals. To investigate the functions of these variant genes, we conducted Gene Ontology (GO) and Kyoto Encyclopedia of Genes and Genomes (KEGG) enrichment analyses. The GO results indicated that the mutated genes were predominantly involved in processes such as microtubule-based movement and cell–cell adhesion (Figure 2) (Table S4), while KEGG analysis showed a concentration in pathways like complement and coagulation cascades and base excision repair (Figure 3) (Table S5). These pathways are closely associated with the animal’s stress resistance, suggesting that mutations in these genes may enhance the stress resistance of Kongshan Cattle.

## 4. Discussion

Kongshan Cattle, historically known as “Bashan cattle”, originate from the northern alpine regions of Tongjiang County, Bazhong City, Sichuan Province, at the southern extremity of the Daba Mountains [1]. These cattle exhibit strong resilience and utility, traits developed through centuries of domestication by local farmers from wild ancestors in the Daba Mountains. Despite their historical significance, the Kongshan Cattle population has experienced a significant decline recently, underscoring an immediate need for breed protection. A survey identified four primary factors contributing to this decline [1]. Firstly, the local cattle industry generally sees lower breeding efficiency compared to beef cattle, leading farmers to sell calves and cows at reduced prices in pursuit of short-term market benefits. Secondly, existing conservation areas and farms have not been fully effective, struggling with inadequate personnel, funding, and imperfect conservation mechanisms. Thirdly, the absence of a dedicated breeding base for Kongshan Cattle means that sustaining the breed through local resources is not feasible. Lastly, the breed’s unique advantages and characteristics have been ineffectively developed or marketed, resulting in inadequate market presence [1]. Given these challenges, conducting in-depth research and analyses of their genetic diversity and structure is imperative to ensuring the preservation and revitalization of this valuable local breed.

Rapid advancements in high-throughput sequencing technologies have fundamentally transformed the study of population genetics across both model and non-model species [12]. With the decreasing costs of sequencing and the completion of livestock genome sequencing projects, whole-genome resequencing has emerged as a crucial tool for investigating genetic variations in livestock. This technique provides a wealth of genetic variation information, including single nucleotide polymorphisms (SNPs) and insertions/deletions (InDels), creating a genomic information repository for exploring livestock phenotypic traits and genetic improvements, thus facilitating in-depth research and utilization of livestock genetic resources [13,14,15]. For instance, Chugang Mei et al. conducted whole-genome resequencing on six phenotypically and geographically diverse domestic Chinese cattle breeds (Qinchuan, Nanyang, Luxi, Yanbian, Yunnan, and Leiqiong cattle), as well as two non-Chinese breeds (Japanese Black and Red Angus cattle). They discovered that the level of genetic variation in Chinese cattle depends on the degree of indicine content and identified many potential selective sweep regions related to breed-specific characteristics, including genes associated with coat color and meat production/quality [16]. Similarly, Xiwen Guan et al. analyzed Dabieshan Cattle from China and detected candidate genes related to fertility, feed efficiency, immune response, heat resistance, and coat color through selective sweeps [17]. Assessments of genomic diversity and signatures of selection using whole-genome sequencing data have also been performed by Xiaoting Xia on Jiaxian Red cattle and Xiaohui Ma on Bohai Black cattle [18,19].

Whole-genome resequencing allows researchers to discover a vast array of genetic variants. For instance, resequencing of the globally renowned Limousin cattle breed identified a total of 13,943,766 variants, including 311,852 bi-allelic SNPs and 92,229 indels [20]. Similarly, resequencing of the famous Holstein dairy cattle breed revealed 365,169 indels [21]. In our study of Kongshan Cattle, the high-quality data acquired after initial quality control showed ratios of Q20 and Q30 bases above 90% and a GC content around 50%, indicating a low error rate in base recognition during sequencing and a high feasibility of the experimental results. This thorough resequencing approach yielded an average of 14,058,387 SNPs per sample. We identified 1,818,975 sites with fragment insertions and 2,987,128 sites with deletions, indicating high genetic diversity within the Kongshan breed. Of these, the SNP analysis revealed 9,957,241 transition-type SNPs and 4,101,146 transversion-type SNPs, resulting in a transition-to-transversion ratio of 2.42. Quality control filtering further highlighted the significant numbers of these variants, especially in coding regions. Like Holstein cattle, where most indels (97.96%) were less than 10 bp with a decreasing trend in length [21], many indels in Kongshan Cattle were also less than 10 bp, although a notable number exceeded 10 bp. The higher proportion of homozygous SNPs suggests a significant genomic divergence between the sampled Kongshan Cattle and the reference genome, underscoring potential unique evolutionary adaptations.

Mutations in coding regions can significantly alter gene functions. Typically, GO and KEGG analyses are employed to explore the functionality of these variant genes. For instance, studies involving whole-genome resequencing of Holstein cattle identified key pathways and genes related to lipid synthesis, such as ACSBG2, which catalyzes the conversion of fatty acids, including long-chain and very-long-chain fatty acids, into their active form, acyl-CoAs, for cellular lipid synthesis [21]. Another example is Glucose 6-phosphate dehydrogenase (G-6PDH), the primary enzyme in the pentose phosphate pathway. This pathway serves as an alternative route for glucose metabolism, producing NADPH necessary for fatty acid synthesis and ribose residues for nucleotide and nucleic acid biosynthesis [21]. In our study, KEGG analysis highlighted significant activity in pathways like complement and coagulation cascades and base excision repair (BER). Research has shown that the complement and coagulation systems are two interconnected protein cascades in plasma, playing crucial roles in host defense and hemostasis, respectively. The activation of the complement system on bacteria supports cellular immune responses and leads directly to bacterial destruction via the formation of the Membrane Attack Complex (MAC) [22]. Increasing evidence suggests that cross-talk between these pathways can rapidly amplify their responses, potentially leading to extensive and prolonged systemic inflammation [23]. Base excision repair is critical for correcting DNA damage caused by oxidation, deamination, and alkylation. The essential role of BER has been underscored by studies showing the inactivation of critical proteins involved in its steps [24]. Notably, the major AP endonuclease in mammalian cells, APE1 (also known as HAP1 and Apex), is vital for survival [25]. This enzyme carries out both AP endonuclease activity and a redox function that are essential for activating several transcription factors and protecting against oxidative stress [26]. These findings are consistent with the observed stress resilience in Kongshan Cattle. The cattle’s resistance may be enhanced by these genetic variations, suggesting that Kongshan Cattle possess robust mechanisms to counter environmental and physiological stresses.

## 5. Conclusions

In this study, we uncovered significant genomic diversity within Kongshan Cattle, demonstrated by an average of 14 million SNPs per sample, substantial heterozygosity, and numerous insertions and deletions impacting gene functionality. Our analysis pinpointed genes linked to stress resistance, underscoring potential adaptive traits that bolster the breed’s resilience. These genetic discoveries not only emphasize the critical need to protect the genetic diversity of Kongshan Cattle as their populations decline but also bolster efforts to stabilize their genetic lineage.

## Figures and Tables

**Table 1:** Basic information on the resequencing data quality for Kongshan Cattle.

Sample_ID	Clean_Reads	Clean_Base	Q20 (%)	Q30 (%)	GC (%)
1	61,784,587	1.68 × 10^10^	98	94.74	43.08
2	59,958,158	1.595 × 10^10^	97.74	93.88	43.31
3	62,129,604	1.711 × 10^10^	98.01	94.82	42.91
4	65,090,871	1.812 × 10^10^	97.82	94.3	42.99
5	66,905,586	1.872 × 10^10^	97.87	94.38	42.53
6	60,576,373	1.618 × 10^10^	98.05	94.64	42.33
7	65,663,649	1.853 × 10^10^	97.67	93.89	42.36
8	65,313,818	1.816 × 10^10^	98.14	95.09	42.59
9	63,172,736	1.729 × 10^10^	98.18	95.17	43.14
10	67,870,038	1.926 × 10^10^	97.5	93.58	42.93
11	64,066,177	1.768 × 10^10^	97.91	94.79	43.17
12	63,900,471	1.764 × 10^10^	97.65	94.27	43.23
13	62,175,800	1.68 × 10^10^	97.71	93.78	42.58
14	57,245,601	1.5 × 10^10^	98.01	94.83	43.21
15	60,967,171	1.681 × 10^10^	97.82	94.4	43.11
16	64,245,777	1.754 × 10^10^	97.84	94.26	41.55
17	63,357,352	1.742 × 10^10^	98.12	94.93	42.7
18	64,428,527	1.791 × 10^10^	97.55	93.76	41.47
19	63,561,727	1.766 × 10^10^	97.76	94.11	42.17
20	54,372,159	1.553 × 10^10^	96.59	91.17	42.34
21	68,638,135	1.918 × 10^10^	98.11	95.15	43.45
22	66,325,088	1.851 × 10^10^	98.02	94.76	42.65
23	59,818,079	1.583 × 10^10^	97.94	94.59	43.61
24	66,007,821	1.841 × 10^10^	98.1	94.9	42.39
25	65,473,203	1.81 × 10^10^	98.23	95.32	42.82
26	64,494,351	1.746 × 10^10^	98.28	95.29	42.44
27	62,882,217	1.712 × 10^10^	97.96	94.62	42.93
28	67,728,437	1.887 × 10^10^	97.92	94.67	43.19
29	63,606,370	1.737 × 10^10^	97.87	94.17	42.75
29	63,606,370	1.737 × 10^10^	97.87	94.17	42.75
30	60,535,241	1.669 × 10^10^	97.41	93.11	42.45
31	63,851,243	1.742 × 10^10^	98.05	94.66	42.73
32	62,988,034	1.698 × 10^10^	98.13	94.98	41.81
33	64,991,465	1.811 × 10^10^	97.96	94.6	42.73
34	67,136,272	1.887 × 10^10^	97.78	94.15	42.83
35	65,230,266	1.813 × 10^10^	97.92	94.5	42.99
36	58,689,859	1.546 × 10^10^	98.06	94.82	43.41
37	60,724,316	1.638 × 10^10^	98.13	95.07	43.13
38	64,944,314	1.822 × 10^10^	97.85	94.26	42.53
39	68,160,352	1.93 × 10^10^	97.91	94.57	43.04
40	62,904,029	1.747 × 10^10^	97.72	94.08	40.95
Mean	63,547,882	1.75 × 10^10^	97.88	94.43	42.71

**Table 2:** Statistical information on SNP-related indicators for the Kongshan Cattle population.

Sample_ID	SNP Number	Transition	Transversion	Ti/Tv	Heterozygosity	Homozygosity	Hetratio
1	14,207,746	10,064,021	4,143,725	2.42	3,383,344	10,824,402	23.81%
2	13,240,910	9,379,967	3,860,943	2.42	2,774,942	10,465,968	20.95%
3	13,917,295	9,861,512	4,055,783	2.43	3,089,436	10,827,859	22.19%
4	14,023,735	9,933,509	4,090,226	2.42	3,187,450	10,836,285	22.72%
5	14,218,607	10,070,821	4,147,786	2.42	3,418,603	10,800,004	24.04%
6	13,284,189	9,399,724	3,884,465	2.41	2,774,123	10,510,066	20.88%
7	14,454,668	10,243,981	4,210,687	2.43	3,313,682	11,140,986	22.92%
8	13,665,815	9,670,487	3,995,328	2.42	2,993,846	10,671,969	21.90%
9	13,796,846	9,777,019	4,019,827	2.43	3,157,604	10,639,242	22.88%
10	14,330,750	10,154,645	4,176,105	2.43	3,541,413	10,789,337	24.71%
11	14,096,969	9,984,328	4,112,641	2.42	3,306,860	10,790,109	23.45%
12	14,029,291	9,938,041	4,091,250	2.42	3,173,186	10,856,105	22.61%
13	14,046,698	9,950,937	4,095,761	2.42	3,354,423	10,692,275	23.88%
14	13,623,689	9,657,310	3,966,379	2.43	3,238,142	10,385,547	23.76%
15	13,893,159	9,841,825	4,051,334	2.42	2,561,481	11,331,678	18.43%
16	13,861,477	9,800,903	4,060,574	2.41	2,625,420	11,236,057	18.94%
17	13,752,486	9,734,535	4,017,951	2.42	2,886,794	10,865,692	20.99%
18	13,893,767	9,823,070	4,070,697	2.41	3,327,115	10,566,652	23.94%
19	13,958,597	9,887,685	4,070,912	2.42	3,370,129	10,588,468	24.14%
20	13,006,597	9,216,408	3,790,189	2.43	2,850,516	10,156,081	21.91%
21	14,669,911	10,395,409	4,274,502	2.43	3,701,319	10,968,592	25.23%
22	14,672,998	10,394,392	4,278,606	2.42	3,753,107	10,919,891	25.57%
23	13,703,719	9,716,393	3,987,326	2.43	3,042,421	10,661,298	22.20%
24	13,868,570	9,815,622	4,052,948	2.42	3,195,874	10,672,696	23.04%
25	14,100,902	9,991,380	4,109,522	2.43	3,155,358	10,945,544	22.37%
26	14,254,834	10,092,102	4,162,732	2.42	3,558,844	10,695,990	24.96%
27	14,576,978	10,331,589	4,245,389	2.43	3,705,680	10,871,298	25.42%
28	15,117,210	10,711,301	4,405,909	2.43	3,892,154	11,225,056	25.74%
29	14,278,695	10,119,132	4,159,563	2.43	3,468,084	10,810,611	24.28%
30	13,888,549	9,843,688	4,044,861	2.43	3,244,361	10,644,188	23.35%
31	14,177,372	10,040,463	4,136,909	2.42	3,435,967	10,741,405	24.23%
32	14,129,984	9,995,601	4,134,383	2.41	3,403,540	10,726,444	24.08%
33	14,251,061	10,098,826	4,152,235	2.43	3,486,580	10,764,481	24.46%
34	14,305,678	10,138,099	4,167,579	2.43	3,456,719	10,848,959	24.16%
35	14,433,117	10,220,758	4,212,359	2.42	3,579,334	10,853,783	24.79%
36	13,417,037	9,508,151	3,908,886	2.43	2,252,453	11,164,584	16.78%
37	14,195,922	10,055,439	4,140,483	2.42	3,222,503	10,973,419	22.70%
38	14,373,091	10,183,304	4,189,787	2.43	3,692,607	10,680,484	25.69%
39	14,571,879	10,327,152	4,244,727	2.43	3,555,457	11,016,422	24.39%
40	14,044,682	9,920,111	4,124,571	2.4	3,529,280	10,515,402	25.12%
Mean	14,058,387	9,957,241	4,101,146	2.42	3,266,504	10,791,883	23.19%

**Table 3:** Statistical information on small InDel-related indicators for the Kongshan Cattle population.

	CDS					Genome				
Sample	Insertion	Deletion	Homo	Het	Total	Insertion	Deletion	Homo	Het	Total
1	1467	1538	2317	688	3005	757,961	920,833	1,283,930	394,864	1,678,794
2	1358	1414	2165	607	2772	700,447	842,834	1,221,296	321,985	1,543,281
3	1459	1535	2315	679	2994	744,727	898,325	1,282,792	360,260	1,643,052
4	1423	1543	2267	699	2966	748,548	907,041	1,282,660	372,929	1,655,589
5	1408	1514	2207	715	2922	761,246	925,021	1,285,532	400,735	1,686,267
6	1190	1299	1972	517	2489	709,281	855,198	1,237,083	327,396	1,564,479
7	1497	1596	2363	730	3093	776,197	942,360	1,330,308	388,249	1,718,557
8	1292	1356	2072	576	2648	727,736	881,485	1,257,342	351,879	1,609,221
9	1394	1512	2210	696	2906	735,050	889,051	1,255,430	368,671	1,624,101
10	1426	1584	2278	732	3010	765,555	931,497	1,283,993	413,059	1,697,052
11	1474	1564	2350	688	3038	751,905	913,126	1,279,162	385,869	1,665,031
12	1485	1562	2354	693	3047	749,946	909,775	1,286,354	373,367	1,659,721
13	1352	1450	2122	680	2802	748,297	907,088	1,266,272	389,113	1,655,385
14	1438	1455	2279	614	2893	724,695	873,677	1,222,512	375,860	1,598,372
15	1484	1545	2447	582	3029	747,521	899,988	1,344,468	303,041	1,647,509
16	1186	1303	1975	514	2489	752,379	908,377	1,343,068	317,688	1,660,756
17	1382	1409	2156	635	2791	737,837	890,391	1,287,817	340,411	1,628,228
18	1188	1312	1907	593	2500	747,742	909,314	1,261,195	395,861	1,657,056
19	1295	1386	2059	622	2681	742,907	900,444	1,253,999	389,352	1,643,351
20	1268	1424	2057	635	2692	689,927	832,492	1,195,256	327,163	1,522,419
21	1550	1641	2449	742	3191	784,033	959,608	1,308,286	435,355	1,743,641
22	1479	1615	2304	790	3094	785,104	963,108	1,303,248	444,964	1,748,212
23	1433	1576	2278	731	3009	728,003	880,250	1,255,579	352,674	1,608,253
24	1295	1430	2114	611	2725	742,952	902,636	1,267,881	377,707	1,645,588
25	1463	1545	2305	703	3008	756,507	917,653	1,302,193	371,967	1,674,160
26	1382	1512	2187	707	2894	762,604	932,059	1,272,207	422,456	1,694,663
27	1565	1720	2481	804	3285	776,120	948,553	1,292,698	431,975	1,724,673
28	1658	1796	2568	886	3454	808,491	994,753	1,345,333	457,911	1,803,244
29	1456	1552	2240	768	3008	763,481	928,942	1,286,165	406,258	1,692,423
30	1387	1459	2192	654	2846	741,645	898,622	1,261,476	378,791	1,640,267
31	1385	1514	2209	690	2899	757,424	921,213	1,274,811	403,826	1,678,637
32	1255	1423	2001	677	2678	755,652	923,274	1,276,809	402,117	1,678,926
33	1473	1502	2287	688	2975	762,705	928,432	1,282,863	408,274	1,691,137
34	1444	1534	2276	702	2978	767,042	934,250	1,294,057	407,235	1,701,292
35	1473	1531	2282	722	3004	770,460	939,792	1,289,548	420,704	1,710,252
36	1398	1471	2298	571	2869	717,673	860,972	1,314,189	264,456	1,578,645
37	1482	1578	2337	723	3060	758,186	921,732	1,301,864	378,054	1,679,918
38	1451	1546	2289	708	2997	768,059	936,025	1,271,853	432,231	1,704,084
39	1518	1623	2338	803	3141	778,855	950,563	1,314,358	415,060	1,729,418
40	1218	1337	1926	629	2555	759,965	926,844	1,260,633	426,176	1,686,809
Mean	1405.775	1505.15	2230.825	680.1	2910.925	751,621.625	912,689.95	1,280,913	383,398.575	1,664,311.575

**Table 4:** Statistics of gene types affected by mutations.

Sample	Genes with Non-Synonymous SNP	Genes with InDel
1	14,509	3350
10	14,553	3400
11	14,475	3386
12	14,556	3430
13	14,227	3188
14	14,312	3269
15	14,411	3397
16	13,665	2917
17	14,180	3191
18	13,506	2936
19	14,087	3102
2	14,077	3157
3	14,415	3371
35	14,525	3386
36	14,328	3199
37	14,603	3420
38	14,469	3335
39	14,598	3494
4	14,377	3287
40	13,453	2953
5	14,351	3270
6	13,619	2843
7	14,566	3459
8	13,963	3034
9	14,234	3244
20	13,826	3075
21	14,807	3530
22	14,638	3461
23	14,377	3335
24	14,045	3125
25	14,466	3398
26	14,214	3251
27	14,828	3603
28	15,089	3754
29	14,405	3345
30	14,254	3240
31	14,332	3251
32	13,817	3066
33	14,478	3358
34	14,460	3310
Mean	14,302.38	3278

## Data Availability

The data underlying this article will be shared upon reasonable request to the corresponding author.

## Supplementary Materials

The following supporting information can be downloaded at: https://www.mdpi.com/article/10.3390/ani14213056/s1. Genomic Insights and Conservation Priorities for Kongshan Cattle: A Whole-Genome Resequencing Approach, Table S1: Analysis of All Differential Variants between Samples; Table S2: Analysis of All Differential Small InDels between Samples; Table S3: Overlap of Variant Genes Across 40 Kongshan Cattle Individuals; Table S4: GO Enrichment Analysis of Variant Genes; Table S5: KEGG Enrichment Analysis of Variant Genes.
